# Sex differences in mania phenotype and ethanol consumption in the lateral hypothalamic kindled rat model

**DOI:** 10.1038/tp.2015.30

**Published:** 2015-03-24

**Authors:** O A Abulseoud, N A Gawad, K Mohamed, C Vadnie, U M Camsari, V Karpyak, M A Frye, D-S Choi

**Affiliations:** 1 Department of Psychiatry and Psychology, Mayo Clinic, Rochester, MN, USA; 2 Department of Molecular Pharmacology and Experimental Therapeutics, Mayo Clinic, Rochester, MN, USA

## Abstract

Sex differences have been observed in mania phenotypes in humans. However the mechanisms underlying this difference are poorly understood. Activating the lateral hypothalamus is implicated in manic-like behaviors in rodents. Using newly established lateral hypothalamus kindled (LHK) rat mania model, we investigated sex differences of manic-like behaviors and its correlation with voluntary ethanol intake. We stimulated the lateral hypothalamus bilaterally in the male and female Wistar rats over five consecutive days. We recorded and quantified kindling-induced behaviors for each individual animal. We also assessed ethanol consumption using a two-bottle choice ethanol drinking as well as circadian locomotor activity counts daily throughout the experiment. We found notable sex differences in several aspects of manic-like behaviors during kindling. Males exhibited a significantly increased locomotor activity during the light phase, and reduced rest interval. On the other hand, females displayed significantly higher ethanol consumption and more frequent rearing behavior. However, no sex differences were present in the duration of sexual, feeding or grooming behaviors or in dark-phase activity counts. The excessive alcohol intake in LHK female rats is reminiscent of clinically reported sex differences in bipolar patients while the other phenotypic sex differences such as rearing and locomotor activity are less clearly described in clinical studies. Overall, our results lend further evidence for the validity of the LHK rat as a useful model to study brain region-specific molecular changes during mania and its correlation with alcohol use disorders.

## Introduction

Despite equal prevalence rates of mania in men and women,^1, 2^ a solid body of literature supports the concept of sex differences. Women with bipolar disorder are more likely to be hospitalized for mania^3^ and have higher rates, in comparison with men of mixed mania,^4, 5^ mixed hypomania,^6^ mixed depression^7^ and rapid cycling.^8, 9, 10^ Although the prevalence rates for alcohol use disorders comorbidity are higher for bipolar men compared with bipolar women, the odds ratio of risk is significantly higher for bipolar women in comparison with women in the general population.^11^ However, alcohol comorbidity during hospitalization for mania^12^ and self-report measures of maximum drinks per day^13^ have suggested that bipolar women may have alcohol use patterns that meet significant clinical concern. Bipolar manic men, on the other hand, had earlier onset of first-episode mania,^14^ and were more likely than manic women to exhibit hypersexuality^15^ and to report the presence of ‘problem behaviors' and excitement.^16, 17^

Most of these clinical studies, however, are limited to understand the molecular basis of disease phenotype because of the presence of comorbidities, effects of medications and sometimes the retrospective nature. Using a valid animal model to study these sex differences is a critical step in understanding the neurobiological basis of these differences and to examine the possibility of sex-specific treatment for mania.

The hypothalamus is the brain region that performs the complex task of coordinating various neural systems that mediate different functional responses,^18^ integrating the motivational aspects of behavior with visceral-motor responses, regulating energy homeostasis,^19^ initiating sexual behavior^20, 21, 22, 23^ and synchronizing the rhythms of all biological processes with environmental changes to achieve the optimal adaptive response.^24^ The lateral hypothalamic area in particular has been implicated in the processing of sensory information and the expression of behaviors associated with hunger and thirst, aggression, reproduction^25^ and in mediating general arousal and sensory sensitization associated with motivational behavior.^26^

Several lines of evidence suggest the involvement of the hypothalamus in the pathophysiology of mania. Structural brain imaging studies suggest a dilation of the third ventricle in the region where hypothalamic nuclei are located adjacent to its walls, indirectly implicating reduced hypothalamic volume.^27, 28, 29, 30^ Furthermore, two postmortem studies^31, 32^ showed significant reduction in the volume of the hypothalamic region in patients with bipolar disorder type I compared with those with major depression and healthy controls. Further evidence emerges from neurosurgical case reports of hypothalamic hamartomata where patients with these rare non-neoplastic nodules present with manic-like symptoms described as mood instability, aggressiveness, restlessness and severe rage attacks accompanied by a strange feeling of ‘pressure to laugh' called gelastic seizures.^33,34^ On the other hand, preclinical studies in rats,^35^ pigs^36^ and monkeys^37^ have shown that high frequency stimulation of the hypothalamus engages functional circuits involved in different behaviors reminiscent of mania such as hypersexuality, aggression, increased locomotor activity and disturbed sleep–wake cycle.^38^ However, we have specifically employed the kindling paradigm to facilitate spontaneous firing of the lateral hypothalamic neurons and hence induce manic-like behaviors that can carry over during the poststimulation phase of the experiment. Amygdala kindling has been proposed as a model of epilepsy by Post and Weiss^39^ to explain the efficacy of the anticonvulsant carbamazepine in the treatment of mania in patients with bipolar disorder. However, the symptomatology induced by amygdala kindling was distinct from any caused by mania.^39^ Bilateral lateral hypothalamic lesioning, on the other hand, does not lead to manic-like behaviors, rather it causes severe reduction in mobility, food, water intake and body weight.^40,41,42^ Some of these behaviors could arguably be reminiscent of the depressive phase of bipolar illness. Because either hypo-function of hypothalamus in humans or hyper-function of hypothalamus of animal models attribute to bipolar disorders, dysregulation of hypothalamus is implicated in bipolar disorders collectively.

We have recently developed a valid rat model for mania through lateral hypothalamic kindling (LHK).^38^ Our model displayed significantly increased sexual self-stimulation excessive rearing, feeding and grooming during the kindling interval. Moreover, the LHK male rats also drank more ethanol during the mania-induction days compared with baseline ethanol consumption levels and exhibited increased total locomotor activity with reduced rest interval during the mania-induction and post-mania days compared with baseline activity levels and rest intervals. In addition, chronic administration of lithium or valproic acid significantly attenuated manic-like behaviors in the LHK rat model.^38^ In this study, we focused on sex differences in manic-like behavioral phenotype and voluntary ethanol consumption in rats. Although it is difficult to establish the link between human and rodent behaviors, we have looked at sex differences in feeding behavior in the LHK rat model because binge eating disorder is the most common eating disorder in the United States, with a lifetime prevalence of ~3.5% in adult women, 2.0% in adult men.^43^ In addition, a high rate of comorbidity between bipolar disorder and eating disorders has been shown both in community samples and clinical studies.^44, 45^ Similarly, we also measured changes in grooming behavior as a putative marker of stress and anxiety^46, 47^ for two reasons, first manic episode is a period of intense stress associated with activation of hypothalamic–pituitary–adrenal axis stress hormones,^48^ second, the comorbidity between mania and anxiety disorders is common^49^ specifically among women.^50^

## Materials and methods

**Figure 1:**
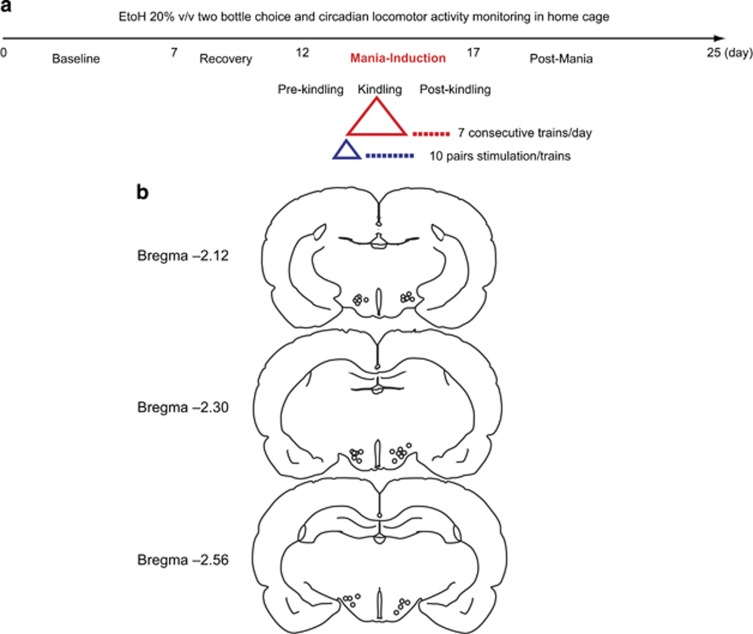
(**a**) Study design: baseline phase for 7 days, followed by surgical implantation of stimulating electrode and recovery for 5 days before mania induction took place for 5 days. Each day, the animal was allowed an initial pre-kindling habituation period (pre-kindling) for 30 min followed by kindling phase consisting of seven consecutive trains starting at 1 V for the first train then increased to 2 V for the subsequent six trains. Two-minute rest intervals were allowed between trains. Each train consisted of 10 pairs of stimulation (10 s) alternating with rest (30 s). The animal remained in the monitored cage for 30 min during the post-kindling period before it was returned back to home cage for 7 days (post-mania days). Locomotor activity counts and voluntary ethanol (EtOH) consumption were monitored continuously throughout the study period. (**b**) An illustration of stimulating electrode tip locations plotted within the dorso-medial part of the LHA regions. AP coordinates were between −2.12 to −2.56 from bregma according to Paxinos Atlas. AP, anteroposterior; LHA, lateral hypothalamic area.

All experimental procedures were approved by the Mayo Clinic Institutional Animal Care and Use Committee. In this study, we have used the same methods detailed in our first published mania-induction experiments.^38^ (Figure 1a illustrates the study design).

### Animals

The experiments were done on adult male and female Wistar rats (*n*=12 per group, age 12–16 weeks, weight 250–300 g at the beginning of experiment) obtained from Charles River Laboratories International (Wilmington, MA, USA). The rats were housed in individual cages on a 12-h light/dark cycle (lights on at 0600 h), free supply of food (*ad libitum*) and tap water.

### Surgical implantation of stimulating electrodes

Anesthetized animals (isoflurane inhalation: 3.0% during induction and 1–1.5% during maintenance) were secured in the stereotaxic apparatus (David Kopf Instruments, Tujunga, CA, USA) and the skull leveled between bregma and lambda. Bipolar stimulating electrodes (#MS303 twisted stainless steel, outer diameter 125 μm, Plastics One, Roanoke, VA, USA) were implanted bilaterally into the lateral hypothalamic area (anteroposterior: −2.28, mediolateral: ±2.7, dorsoventral: −8.5 mm from skull surface). Electrodes were implanted with a 7º angle to allow enough room to attach the stimulating cords on both sides. Electrodes were secured to the skull using dental cement and three screws. Animals were closely observed over 5 days post surgery. Animals with any neurological signs of brain damage were excluded from the study.

### Mania-induction procedure

All the procedures took place in the early light phase between 0600 h and 0800 h during mania-induction days. The animal was placed individually into a clear Plexiglas cage (12'' × 12” × 30”, with ordinary bedding and food pellets on the floor) for 30 min at the beginning of the experiment to habituate to the new environment. This interval was called the pre-kindling interval and was followed by 60 min of kindling using the following stimulation parameters: bipolar configuration, 180 Hz s^−1^ frequency, 200 μs pulse width and 10-s pulse durations followed by 30 s of rest. Seven trains were applied, each consisting of 10 pairings of 10-s duration stimulation pulses alternating with 30 s of rest, and 2 min of rest were allowed between trains. Stimulation amplitude was started at 1 V and was increased to 2 V for the rest of the stimulation trains. The reason we used only 1–2 V and did not escalate stimulation amplitude to 7 V as we have done in the first experiment is that we wanted to observe phenotypic differences between males and females at lower stimulation threshold and using higher amplitude could mask these subtle differences by giving a celling effect. Following all the seven kindling trains, the animal was kept in the observation chamber for 30 more minutes as post-kindling interval. Subsequently, the stimulating cord was disconnected and the animal was returned back to the home cage. The exact same procedure was repeated for five consecutive days. We used 5 days to span throughout the average cycle length, which typically lasts 4–5 days^25^ to avoid the confounding of hormonal variability during the estrous cycle phases. Furthermore, kindling-induced manic-like behavior is, by itself a stressor that could potentially perturb the hypothalamic-pituitary gonadal axis function as evident by abnormal gonadal hormone levels during mania.^51, 52^

### Monitoring and quantifying behaviors during the process of mania-induction

Continuous video recording of behaviors was performed over the 2-h mania-induction session. Recorded video files were transferred and stored in two separate external hard drives. Each animal recording was reviewed independently and behaviors were coded separately by two trained masked raters (UMC and NAG), and target behaviors were reviewed by the principal investigator of the study (OAA). The four primary behaviors were coded: (1) sexual behavior: the duration (in 5-s increments) of any oral contact with the genital area or immediate perigenital area or darting in female rats (Supplementary Videos S1, S2, S3); (2) rearing behavior: the number of episodes of standing on the two hind limbs for more than 5-s duration (Supplementary Video S4); (3) grooming behavior: the total time (in 5-s increments) of repeated mouthing of any body part except the genital and perigenital areas or repeated movement of the fore or hind paws over the snout, face, head, trunk or tail (Supplementary Video S5); and (4) feeding behavior: the duration (in 5-s increments) where the animal is observed holding and chewing food pellets. Chewing without food or chewing bedding was not considered feeding behavior (Supplementary Video S6).

### 24-hour sleep–wake cycle and locomotor activity monitoring in home cage during baseline, mania-induction and post-mania days

Circadian locomotor activity counts in home cage were recorded using an infrared motion detector interfaced with a computerized data acquisition system (ClockLab, ActiMetrics, Wilmette, IL, USA) and later analyzed using MATLAB (The MathWorks, Natick, MA, USA). Total locomotor activity counts (bout analysis) and total activity time for light and dark phases were measured separately for each day of the experiment during baseline, mania-induction days and post-mania days. The nonactivity time was calculated by subtracting total activity time (minutes) from 720 min (12-h light/dark phase). This outcome measure was used as a surrogate marker for rest or sleep.

### Voluntary ethanol consumption via two-bottle choice paradigm

The animals were offered 20% (v/v) ethanol vs tap water in a two-bottle choice paradigm throughout the experiment. Ethanol and water consumption were measured by weighing the animal and the two bottles daily. The amount of ethanol and water consumed were determined by subtracting the bottle weights from their initial weight. To avoid the confounding of side preference, we switched the bottle side every time fluid measurements were taken.

### Euthanasia and brain histology for verification of electrode tip location

The animals were lightly anesthetized in a CO_2_ chamber and euthanized by rapid decapitation. The brain was carefully collected and fixed in paraformaldehyde solution for 24 h followed by 30% sucrose solution for 1 week, then covered with optimal cooling temperature compound for cryostat sectioning (Ted Pella, Redding, CA, USA) and stored at −80 °C until histology was performed. The brains were sectioned on a cryostat (50 μm) and stained with hematoxylin and eosin. Verification of electrode tip location was done according to the atlas of Paxinos and Watson.^53^ (Electrode location is illustrated in Figure 1b).

### Statistical analysis

Behavioral variables were presented as means±s.e.m. Four manic-like behaviors (duration of sexual, grooming and feeding behaviors, and frequency of rearing behavior) were quantified at three time intervals (pre-kindling, kindling and post-kindling) during each day of the five mania-induction days and summed together. Ethanol drinking (g kg^−1^ per day) and locomotor activity (counts per day) were also presented as means±s.e.m and summed into three time intervals (baseline days, mania-induction days and post-mania-induction days). To correct for potential individual variability at baseline, we calculated behavioral changes from baseline to kindling (or mania induction) and to post-kindling (or post-mania), then we used a repeated measures analysis of variance (ANOVA) to examine for differences between males and females in individual behavioral manifestations during each time interval. Pearson correlation was performed to examine for potential relationship between the amount of ethanol (g kg^−1^ per day) consumed during the mania-induction days and locomotor activity counts. When a significant interaction was found, a *post hoc* testing was performed to determine pair-wise differences. Results were considered significant where *P*<0.05.

## Results

### Phenotypic differences in LHK-induced sexual and rearing behaviors between male and female rats

**Figure 2:**
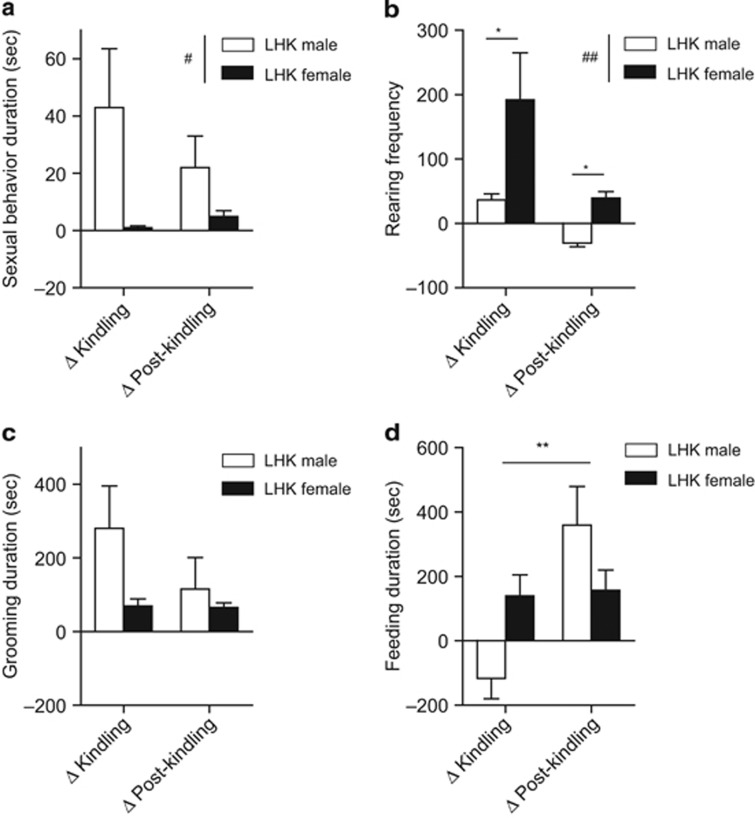
Change in manic-like behaviors: from the pre-kindling to kindling (Δkindling) and post-kindling (Δpost-kindling) were compared between LHK male and female rats. (**a**) A nonsignificant trend towards a main effect of sex (F_1,18_=4.34, ^#^*P*=0.0516) was observed in the duration of sexual behavior, and significant main effects of kindling (F_1,20_=12.15, ^*^P=0.002) and sex (F_1,20_=9.74, ^##^*P*=0.005) were evident in rearing behavior (**b**), whereas no significant effect for kindling or sex were detected in grooming behavior (**c**). However, a significant main effect of kindling (F_1,20_=6.13, ^**^P=0.02) and an interaction between kindling and sex (F_1,20_=5.29, *P*=0.03) were evident in the duration of feeding behavior (**d**) by repeated measures ANOVA. ^*^P<0.05 by Tukey *post hoc* test; *n*=9–10 per rat type. Data are expressed as mean±s.e.m. ANOVA, analysis of variance; LHK, lateral hypothalamus kindled.

During the surgical procedure, five animals (two males and three females) were excluded due to electrode location outside target area (*n*=2) or pulling stimulating electrode (*n*=2) or dying during surgery (*n*=1) among 24 animals (12 males and 12 females) used in this experiment. Analysis of LHK-induced mania-like behaviors revealed notable sex differences in rearing behaviors, whereas no differences were found in sexual, grooming and feeding behaviors. For sexual behavior (Figure 2a), male rats showed a nonsignificant trend toward increased sexual behavior compared with female rats. Repeated measures ANOVA yielded a trend towards a main effect of sex (F_1,18_=4.34, *P*=0.0516), but not kindling (F_1,18_=0.99, *P*=0.33) nor interaction (F_1,18_=2.1, *P*=0.16).

Since the rearing behavior is correlated with manic-like behaviors,^54, 55^ we measured a number of episodes of standing on the two hind limbs for more than 5 s (Figure 2b). The change in the frequency of rearing behavior from pre-kindling to kindling and post-kindling showed significant main effect of kindling (F_1,20_=12.15, *P*=0.002), sex (F_1,20_=9.74, *P*=0.005), but not interaction (F_1,20_=1.86, *P*=0.18). Compared with males, females had significantly more rearing during kindling (males vs females: 36.5±9.2 vs 192.0±72.6) and post-kindling (males vs females: −30.2±6.0 vs 39.4±9.8).

We observed no significant effect of sex (F_1,20_=1.71, *P*=0.2), kindling (F_1,20_=2.67, *P*=0.1) or an interaction between the two factors (F_1,20_=2.44, *P*=0.1) on the duration of grooming behavior by repeated measures ANOVA (Figure 2c). However, a significant main effect of kindling (F_1,20_=6.13,*P*=0.02), but not sexes (F_1,20_=0.15,*P*=0.7), with a significant interaction between kindling and sex (F_1,20_=5.29,*P*=0.03) was evident in the duration of feeding behavior (Figure 2d), suggesting that during the mania-induction phase, male rats may have suppressed appetite, which is consistent with clinical reports.^56^ However, further experiments are needed to explore this speculation as we only measured food intake during mania induction.

### LHK female rats consume significantly more ethanol compared with LHK male rats

**Figure 3:**
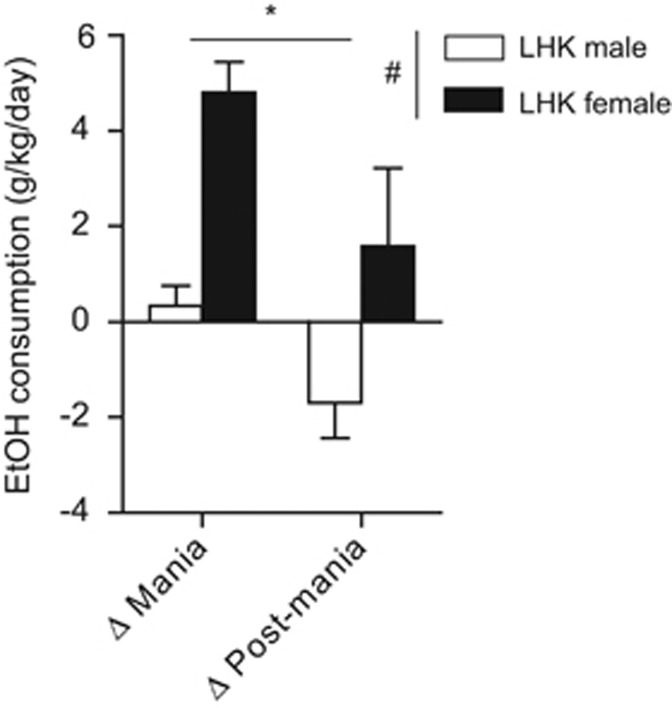
Ethanol (EtOH) consumption (g kg^−1^ per day) showed significant main effect for kindling (F_1,17_=4.93, ^*^P=0.04) and sex (F_1,17_=10.79, ^#^*P*=0.004) by repeated measures ANOVA. ^*^P<0.05 by Tukey *post hoc* test; *n*=9–10 per rat type. Data are expressed as mean±s.e.m. ANOVA, analysis of variance; LHK, lateral hypothalamus kindled.

Next, we examined whether LHK-induced mania promote alcohol consumption as it is well known that mania increases a risk of alcohol abuse or binge alcohol consumption.^11^ Interestingly, female rats consumed significantly more ethanol compared with male rats during the mania (Figure 3). Repeated measures ANOVA on the dependent variable ethanol drinking revealed significant main effects for mania induction (F_1,17_=4.93, *P*=0.04) and sex (F_1,17_=10.79, *P*=0.004), but no interaction between the two factors (F_1,17_=0.25, *P*=0.62). Female rats drank significantly more ethanol than males during mania-induction days (males vs females: 0.3±0.4 vs 4.7±0.6, g kg^−1^ per day, *P*=0.002) and a trend during post-mania-induction days (males vs females: −1.7±0.7 vs 1.5±1.6, g kg^−1^ per day, *P*=0.08).

### LHK male rats exhibit more hyperactivity and reduced rest intervals than LHK female rats

**Figure 4:**
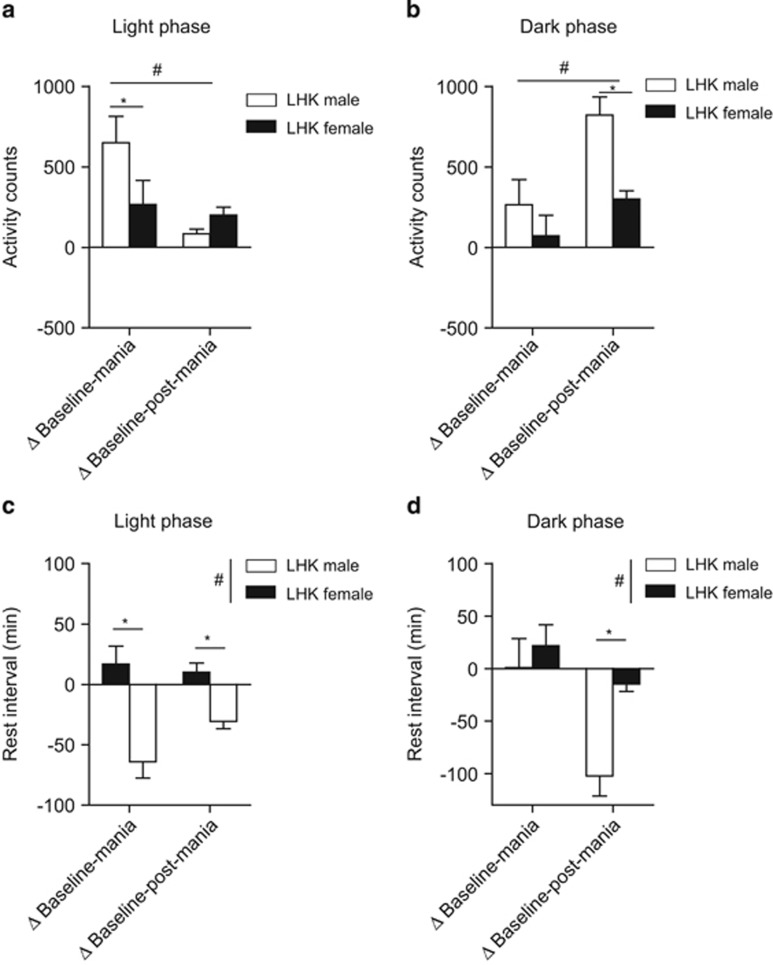
Changes in circadian locomotor activity counts from baseline to mania-induction days (Δmania) and to post-mania-induction days (Δpost-mania) were compared between LHK male and female rats in both the light and dark phases. (**a**) Shows changes in light phase activity counts. Significant main effect for mania induction (F_1,12_=12.56, ^*^P=0.004) and an interaction between mania induction and sex (F_1,12_=7.82, ^#^*P*=0.01) were evident. The changes in dark-phase activity counts (**b**) show significant main effect of mania induction (F_1,14_=11.89, ^#^*P*=0.003) and sex (F_1,14_=9.85, ^*^P=0.007). Changes in light-phase rest interval (**c**) showed significant effect of sex (F_1,14_=30.44, ^#^*P*<0.0001) with males exhibiting significantly less rest intervals during both mania induction (^*^P=0.01) and post-mania-induction days (^*^P=0.002). Dark-phase rest interval (**d**) showed significant effect of sex (F_1,14_=8.57, ^#^*P*=0.01) and mania induction (F_1,14_=14.02, ^*^P=0.002), by two-way ANOVA; ^*^P<0.05 by Tukey *post hoc* test; *n*=9–10 per rat type. Data are expressed as mean±s.e.m. ANOVA, analysis of variance; LHK, lateral hypothalamus kindled.

As manic-like behaviors alter circadian patterns or dysregulated circadian can influence mania phenotype,^57^ we investigated if manic-like behaviors are altered in light and dark phases (Figure 4). Light phase activity counts had a significant main effect for mania induction (F_1,12_=12.56, *P*=0.004) in all animals without differences between male and female rats (F_1,12_=2.26, *P*=0.15) but with a significant interaction between mania induction and sex (F_1,12_=7.82, *P*=0.01) by repeated measures ANOVA. Males showed more change (from baseline) in light phase activity compared with females during mania-induction days: (males vs females: 649.9±163.4 vs 225.2±93.79, *P*=0.04), but not during post-mania-induction days (males vs females: 84.60±27.06 vs 199.8±49.31, *P*=0.1; Figure 4a).

Similarly, the change in dark phase activity counts showed significant main effect of mania-induction (F_1,14_=11.89, *P*=0.003) and sex (F_1,14_=9.85, *P*=0.007) but not interaction between the two factors (F_1,14_=2.06, *P*=0.17). Males were significantly more active than females during post-mania-induction days (males vs females: 821.2±113.2 vs 300.0±52.0, *P*=0.003), but not during mania-induction days (males vs females: 264.9±155.0 vs 70.7±127.3, *P*=0.2; Figure 4b).

Light phase rest interval showed significant effect of sex (F_1,14_=30.44, *P*<0.0001), but not mania induction (F_1,14_=1.46, *P*=0.24) or interaction (F_1,14_=3.31, *P*=0.09). Males had significantly less rest intervals during both mania-induction (males vs females: −64.0±13.5 vs 16.9±14.7 min, *P*=0.01) and post-mania-induction days (males vs females: 84.6±27.0 vs 10.2±7.4 min, *P*=0.002; Figure 4c). On the other hand, a significant effect of sex (F_1,14_=8.57, *P*=0.01) and mania-induction (F_1,14_=14.02, *P*=0.002), without interaction between the two factors (F_1,14_=3.22, *P*=0.09) was observed by repeated measures ANOVA in the dark phase. Males had significantly less rest intervals during post-mania-induction days (males vs females: −102.3±18.9 vs −14.5±7.2, *P*=0.002), but not during mania-induction days (males vs females: 0.6±27.7 vs 21.7±19.8, *P*=0.5; Figure 4d).

## Discussion

Our findings demonstrate that in the LHK mania model, females exhibited similar manic-like behaviors to male rats with certain phenotypic differences in rearing behavior, ethanol consumption and locomotor activity and rest patterns. LHK female rats exhibited solicitation sexual behavior such as darting^58^ as well as active genital licking during kindling and post-kindling (Figure 2a and Supplementary Video S2).

Our results are in agreement with the very limited clinical data examining the sex differences in humans with bipolar disorder.^14, 15, 17^ LHK males in our study exhibited a nonsignificant trend towards more sexual behavior than females during kindling and post-kindling. In addition, the sexual behavior in both males and females was not evoked by an interaction with a rat of the opposite sex. The display of sexual solicitation behavior by LHK females in the form of receptive posture, lordosis and darting,^58^ and in the absence of sexually active male is indicative of maximal sexual motivation.^59^ This behavioral aspect in the LHK female rat is reminiscent of hypersexuality in humans during a manic episode. Young *et al.*^15^ studied 149 acutely manic patients (Young Mania Rating Scale ⩾20) and examined sex differences in individual item scores. Males had significantly higher scores compared with females.

Importantly, the LHK female rat had significant increase in rearing behavior during kindling and post-kindling compared with male rats (Figure 2b and Supplementary Video S4). Rearing behavior is considered a measure of active exploratory behavior.^60^ Change in rearing frequency has been considered a measure for manic-like behavior in animal models.^54, 55^ Female Wistar rats generally display more rearing activity than males at baseline.^61^ To correct for this baseline difference, we compared the changes in rearing frequency rather than actual frequency between males and females.

Interestingly LHK males exhibited significantly more locomotor activity and reduced rest interval during the light phase compared with females (Figures 4a and c). This is contrary to the normal behavior of female rats as more active than males.^62^ It is difficult to speculate the cause of the lesser increase in activity in LHK female rats; however, it may be related to the difference in the chronic mild stress evoked by the single housing condition between males and females (reviewed by Simpson and Kelly^63^) or perhaps by the differences in ethanol consumption.

Compared with LHK males, female rats consumed significantly more ethanol during mania-induction and post-mania days (Figure 3). To determine whether the reduced activity in LHK ‘manic' females is related to their ethanol consumption, we performed a Pearson correlation between daily ethanol consumption and activity counts during mania-induction days. No significant correlation (*r*=−0.246, *P*=0.4) was observed. This finding is consistent with the clinical reports. Strakowski *et al.*^12^ studied 41 acutely manic patients during first hospitalization and found that women were 6.4 times more likely to have a history of comorbid substance use disorder. Similarly, in our previous publication,^13^ there was a small cohort (*n*=27) of alcohol-treatment-seeking women with bipolar disorder. Women reported higher lifetime maximum number of drinks in 24 h (21±11.5) than men (13.4±8.6). Along the same lines, Frye *et al.*^11^ compared the rates of ‘lifetime alcoholism' in a bipolar sample with rates in the general population. The risk of having alcoholism was greater for women with bipolar disorder (odds ratio=7.53) than for men with bipolar disorder (odds ratio=2.77). It is important to mention that the prevalence of alcohol abuse or dependence is significantly higher in men compared with women,^64^ whereas naive Sprague Dawley female rats generally drink more ethanol.^65,66^^67^ Female Wistar rats, on the other hand, were reported to drink less ethanol compared with male rats,^68^ which was attributed to a twofold higher hepatic alcohol dehydrogenase enzyme activity^69^ in the female Wistar rats. Clinically, a study in Ashkenazic Jewish American college students reported that individuals carrying an alcohol dehydrogenase enzyme allele (ADH1B*2) that codes for an isoform that is one order of magnitude more active than that coded by the usual ADH1B*1 (ref. 70) are also protected against heavy alcohol use and alcoholism.^71^ No genetic vulnerability or risk alleles for alcoholism have been reported in bipolar patients.

Another way to understand the observed sex difference in mania phenotype is to relate it to sexual dimorphism in the hypothalamic structure (reviewed by Madeira and Lieberman^72^) and differences in dopamine levels and turnover in the hypothalamus of female rats.^73^ However, regardless of the underlying mechanisms mediating the sex differences in mania, women with bipolar disorder face serious negative impact compared with men.^74^

We would like to acknowledge several limitations for this study. First, as with all animal models, our phenotype findings may not parallel reported gender differences in patients with bipolar disorder. Second, we used only lower stimulation amplitude (1–2V) to induce manic-like behavior. Female rats are susceptible to amygdala kindling at lower stimulation threshold compared with male rats.^75^ Third, in our first report,^38^ manic-like behaviors were only elicited in LHK rats. Kindling of other brain regions such as the nucleus accumbens or infra-limbic cortex was not associated with the induction of manic-like behavior phenotype. For that reason, we only performed kindling of the lateral hypothalamus in this study. Finally, there is a small possibility of false positive results as we did not correct for multiple comparisons. Despite these limitations, our data provide further validity to the LHK rat as a potentially useful model to investigate the neurobiological basis for this intriguing phenomenon and highlight sex-specific manifestations that should be made in the diagnosis or in the development of individualized treatment options.

## Notes

MAF has received grant support from AssureRx Health, Myriad, Pfizer, National Institute of Mental Health (RO1 MH079261), National Institute of Alcohol Abuse and Alcoholism (P20AA017830), Mayo Foundation; has been a consultant to Janssen Global Services, LLC, Mitsubishi Tanabe Pharma Corporation, Myriad, Sunovion and Teva Pharmaceuticals; has received CME/Travel Support/presentation from CME Outfitters and Sunovian. The remaining authors declare no conflict of interest.
