# Supplementary material for: Sex differences in mania phenotype and ethanol consumption in the lateral hypothalamic kindled rat model
Source: Transl Psychiatry. 2015 Mar 24;5(3):e534–. doi: 10.1038/tp.2015.30 (PMC4354358; doi:10.1038/tp.2015.30)
Supplement: Supplementary Video Legends [file tp201530x7.doc]

**Supplementary Video S1**: Sexual behavior (genital licking) in a female rat.

**Supplementary Video S2**: Sexual behavior (darting) in a female rat

**Supplementary Video S3**: Sexual behavior (genital licking of an erected penis) in a male rat.

**Supplementary Video S4**: Rearing behavior.

**Supplementary Video S5:** Grooming behavior.

**Supplementary Video S6:** Feeding behavior.
